# HydrAd: A Helper-Dependent Adenovirus Targeting Multiple Immune Pathways for Cancer Immunotherapy

**DOI:** 10.3390/cancers14112769

**Published:** 2022-06-02

**Authors:** Amanda Rosewell Shaw, Caroline Porter, Greyson Biegert, Lisa Jatta, Masataka Suzuki

**Affiliations:** 1 Department of Medicine, Section of Hematology/Oncology, Baylor College of Medicine, Houston, TX 77030, USA; rosewell@bcm.edu (A.R.S.); caroline.porter@bcm.edu (C.P.); greyson.biegert@bcm.edu (G.B.); lisa.jatta@bcm.edu (L.J.); 2 Center for Cell and Gene Therapy, Baylor College of Medicine, Texas Children’s Hospital, Houston Methodist Hospital, Houston, TX 77030, USA

**Keywords:** cancer immunotherapy, oncolytic adenovirus, helper-dependent adenovirus, cancer gene therapy

## Abstract

**Simple Summary:**

Solid tumors are highly immunosuppressive and develop multiple inhibitory mechanisms that must be targeted simultaneously for successful cancer immunotherapy. Adenoviral vectors are promising cancer gene therapy vectors due to their inherent ability to stimulate multiple immune pathways. Adenoviruses are well characterized, and their genomes are easily manipulated, allowing for therapeutic transgene expression. Oncolytic adenoviruses are engineered to replicate specifically in malignant cells, resulting in cancer cell lysis. However, oncolytic adenoviral vectors have limited transgene capacity. Helper-dependent adenoviral vectors have been developed with the capability of expressing multiple transgenes through removal of all viral coding sequences. We have developed a helper-dependent platform for cancer immunotherapy and demonstrate expression of up to four functional transgenes. This platform allows us to target tumors with specific inhibitory pathways using our library of immunomodulatory transgenes in a mix-and-match approach for a synchronized cancer immunotherapy strategy.

**Abstract:**

For decades, Adenoviruses (Ads) have been staple cancer gene therapy vectors. Ads are highly immunogenic, making them effective adjuvants. These viruses have well characterized genomes, allowing for substantial modifications including capsid chimerism and therapeutic transgene insertion. Multiple generations of Ad vectors have been generated with reduced or enhanced immunogenicity, depending on their intended purpose, and with increased transgene capacity. The latest-generation Ad vector is the Helper-dependent Ad (HDAd), in which all viral coding sequences are removed from the genome, leaving only the cis-acting ITRs and packaging sequences, providing up to 34 kb of transgene capacity. Although HDAds are replication incompetent, their innate immunogenicity remains intact. Therefore, the HDAd is an ideal cancer gene therapy vector as its infection results in anti-viral immune stimulation that can be enhanced or redirected towards the tumor via transgene expression. Co-infection of tumor cells with an oncolytic Ad and an HDAd results in tumor cell lysis and amplification of HDAd-encoded transgene expression. Here, we describe an HDAd-based cancer gene therapy expressing multiple classes of immunomodulatory molecules to simultaneously stimulate multiple axes of immune pathways: the HydrAd. Overall, the HydrAd platform represents a promising cancer immunotherapy agent against complex solid tumors.

## 1. Introduction

Adenovirus (Ad)-based vectors infect both dividing and non-dividing cells, with wide-ranging cellular tropism, making them especially appealing as gene therapy vectors. In addition, their biology is well understood and, because of this, the Ad genome can be easily modified and capsid proteins can be altered to change the cellular tropism of the vector. The Ad replication machinery can be engineered to restrict viral replication to cancer cells, as in oncolytic viruses, or ablate viral replication for gene transfer purposes. For clinical applications, Ads can be manufactured to large titers and to good manufacturing practice (GMP) standards [1]. These characteristics led to the study and development of Ad-based gene therapies over the past few decades.

While an early Ad gene therapy for a genetic disorder had devastating and ultimately lethal toxicities in a clinical trial [2], more recent clinical trials for cancer demonstrated the safety of Ad-based oncolytic vectors. Currently, Ads are the most commonly used oncolytic vector in cancer gene therapy clinical trials, due in part to their well characterized virology, including intrinsic host immune sensing mechanisms (e.g., toll-like receptors) [1,3].

Oncolytic adenoviruses (OAds) are engineered to selectively replicate in malignant cells leading to direct cancer cell lysis and subsequent release of progeny OAds, which can infect neighboring cells within a tumor, thus resulting in multiple rounds of replication and lysis. Preclinical studies demonstrated the potential effectiveness of OAds, but in clinical trials unarmed OAds failed to produce significant antitumor activity. These trials revealed that, although OAds initiate an immune response through pattern recognition receptors and immunologic cell death [4,5], adaptive immune responses were skewed towards anti-viral rather than necessary anti-tumor responses [6]. “Arming” OAds with immunostimulatory or immunomodulatory transgenes can improve their anti-tumor activity, however, tumors have multiple inhibitory pathways that form the tumor microenvironment (TME) and can exert suppressive effects in vivo [7,8].

OAds require most of the Ad genes to remain intact for efficient replication and lytic function. Due to this, their transgene capacity is limited to approximately 3 kb, restricting the ability of armed OAds to address multiple inhibitory pathways presented by solid tumors simultaneously. To increase the ability of OAd to target the complex TME, we developed a platform, called CAdVEC, that utilizes an OAd to provide direct tumor cell lysis and helper function in situ to a co-infected Helper-Dependent Ad (HDAd) vector that encodes complex transgene expression cassettes [9]. HDAds are ideal gene therapy vectors, as they are devoid of all viral coding sequences, retaining only cis-acting packaging and ITR sequences, and thus providing approximately 34 kb transgene capacity. We previously demonstrated that co-infection of OAd and HDAd (Combined Adenoviral Vectors: CAdVEC) results in cancer cell lysis with expression and subsequent amplification of HDAd encoded transgene expression, regardless of tumor type [9].

We have shown that, like HDAd alone, CAdVEC can deliver multiple transgenes in vivo that stimulate different immune axes to produce additive anti-tumor effects in several models including orthotopic, metastatic, and humanized solid tumor bearing mice. Previously evaluated vectors have included stimulatory cytokines, immune checkpoint blockade, and engager molecules capable of redirecting immune cells to tumor cells [10,11,12,13]. Our promising preclinical data led to the initiation of a clinical trial evaluating the efficacy of CAdVEC expressing IL12p70, an anti-PDL1 minibody and HSV thymidine kinase (HSVtk) as a safety switch at Baylor College of Medicine (ClinicalTrials.gov Identifier: NCT03740256). 

The transgenes in our current clinical vector address two immunomodulatory pathways, with 20 kb transgene capacity remaining. Here, we developed a library of HDAd vectors encoding various classes of complimentary immunomodulatory molecules to further increase the breadth of CAdVEC activity. We demonstrated the ability of these vectors to activate immune cells and exploit the large transgene capacity of the HDAd platform to combine these transgenes into a single vector to independently yet additively activate multiple immune pathways. We call this novel platform HydrAd, as this platform will enable targeting of multiple immune pathways simultaneously, like the many headed mythological Hydra.

## 2. Materials and Methods

### 2.1. Adenoviral Vectors

HDAd without a transgene (HDAd0), HD*eGFP*, HD*cytokine*, HD*aPDL1* minibody, HD*CD19.BiTE*, HD*CD44v6.BiTE*, HD*CD44v6Trio*, HD*tkTrio*, oncolytic adenovirus OAd5/3, and helper viruses HV5, HV5/3, HV5/35 were produced as described previously. [9,10,11,12,13,14,15,16,17].

MIP1α (Invivogen, San Diego, CA, USA), RANTES (Invivogen), 41BBL (Invivogen), ICOSL (Invivogen), OX40L (Invivogen), the minibodies [10] αPD1 (clone: MDX-1106) and αCTLA4 (clone: MDX-101) expression cassettes (EF1 promoter) were separately inserted into the pHDΔ28E4 vector (HD*MIP1α*, HD*Rantes*, HD*41BBL*, HD*OX40L*, HD*ICOSL*, HD*PD1mini*, HD*CTLA4mini,* respectively).

The engager molecules were constructed as previously described [12]; the scFv for the MUC1.BiTE has been described elsewhere [18]; the MUC1.BiKE replaces the anti-CD3 scFv with the anti-CD16 scFv clone NM3E2; FAP.BiTE scFv was taken from clone MO36; the HER2.BiTE scFv was derived from our clinical HER2 chimeric antigen receptor [13]. All expression cassettes (EF1 promoter) were separately inserted into the pHDΔ28E4 vector (HD*MUC1.BiTE*, HD*MUC1.BiKE*, HD*FAP.BiTE*, HD*HER2.BiTE*, respectively).

The MIP1α, IL2 (driven by EF1 promoter), and αPDL1mini (driven by CMV promoter) expression cassettes were cloned into pHDΔ21E4 vector (HD*MIP1Trio*). To generate HD*Tetra*, the MUC1.BiTE driven by NFkB promoter expression cassette was cloned together with the clinical HD*tkTrio* expression cassettes into pHDΔ18E4 vector.

After confirmation of sequence and expression, HD*eGFP* vectors were generated utilizing HV5, or chimeric HVs HV5/3 or HV5/35. All other HDAds were rescued with chimeric helper virus HV5/3 [17].

### 2.2. Cell Lines

The human non-small cell lung carcinoma cell line A549, human head and neck squamous cell carcinoma line FaDu, human prostate cancer cell line PC-3, human breast cancer cell line SUM-159, and the human pancreatic line PANC-1 were obtained from the ATCC (Manassas, VA, USA). Human colorectal carcinoma cell line HCT116 was a generous gift from Masato Yamamoto, University of Minnesota. The human lung fibroblast cell line Tig-3-20 (JCRB0506) was obtained from Sekisui, Xenotech (Tokyo, Japan). HER2 deficient FaDu cells (FaDuHer2^−/−^) were described previously [12]. All cell lines were authenticated utilizing short tandem repeat profiling. Cells were cultured under recommended conditions.

### 2.3. Primary Cells

For the generation of non-transduced T cells (ATCs), virus-specific T cells (VSTs), multi-tumor associated antigen-specific T cells (mTAA-Ts), and NK cells, we obtained peripheral blood from healthy donors through an institutional review board (IRB)-approved protocol at Baylor College of Medicine. Human peripheral blood mononuclear cells (PBMCs) were isolated using Ficoll-Paque Plus according to manufacturer’s instructions (Axis-Shield), Oslo, Norway. For ATCs, PBMCs were activated with OKT3 (1 mg/mL) (Ortho Biotech, Bridgewater, NJ, USA) and CD28 antibodies (1 mg/mL) (Becton Dickinson, Franklin Lakes, NJ, USA) and fed every 2 days, beginning the day after stimulation with media supplemented with 10 ng/mL rIL7 and 5 ng/mL rIL15.

Ad-VSTs were produced as described previously [19]. Briefly, PBMCs pulsed with hexon/penton overlapping peptide libraries with subsequent stimulation with irradiated autologous PHA blasts pulsed with hexon/penton pepmix supplemented with IL7 (10 ng/mL) and IL15 (5 ng/mL). TAA-Ts have been described previously [20]. Briefly, monocyte-derived dendritic cells loaded with TAA pepmixes (SURVIVIN, SSX2, MAGE-A4, PRAME, and NY-ESO) were co-cultured with autologous PBMCs in the presence of 10 ng/mL IL7 and IL12, 5 ng/mL IL15.

The methodology to expand human NK cells has been described previously [21]. Briefly, we co-cultured CD56+ PBMCs (enriched via positive magnetic column selection) with irradiated K562-mb15-41BBL at a 1:10 (NK cell:K562) in G-Rex cell culture devices (Wilson Wolf, New Brighton, MN, USA) in Stem Cell Growth Medium (CellGenix, Freiburg im Breisgau, Germany) supplemented with 500 IU/mL recombinant human IL-2 (NIH).

### 2.4. HDAd Transduction Experiment

Tumor cell lines were seeded in 24-well plates and infected with 100 viral particles per cell (vp/cell) of HD*eGFP*, cells were incubated with virus at 37 °C 1 h then washed with PBS and appropriate culture medium was replaced. Cells were imaged using a fluorescent microscope (Leica, Wetziar Germany) 24 h post infection, then harvested using trypsin, for flow cytometry analysis. Live/dead discrimination was determined via inclusion of 7-aminoactinomycin D (7AAD; BD Pharmingen, San Diego, CA, USA). Cells were analyzed for GFP expression using Gallios flow cytometer with Kaluza software (BD Biosciences, Franklin Lakes, NJ, USA) according to the manufacturer’s instructions.

### 2.5. Cytokine/Chemokine ELISA Assay

Forty thousand cells per well were seeded into 24-well plates. Cells were infected with HDAds, OAd, or CAd (OAd:HDAd = 1:1) at the doses indicated in the figure legends and incubated at 37 °C for 48 h. MIP1α and RANTES levels in media were measured using a BD cytokine multiplex bead assay according to manufacturer’s instructions (BD Biosciences). IL-15, IL-2, IL-12p70 levels in media were measured using DuoSet ELISA systems according to manufacturer’s instructions (R&D Systems, Minneapolis, MN, USA).

### 2.6. Minibody ELISA Assay

The minibody binding ELISA assay has been described previously [10]. Briefly, Immulon 2 high binding 96-well plate (VWR, Radnor, PA) was coated with 500 ng/well of recombinant human PD-L1, PD-1, or CTLA-4 (BioVision, Milpilas, CA, USA). After blocking plate with PBS-T containing 3% BSA, serially diluted media containing minibody or media from GFP-transfected cells were added and incubated at 4 °C for 24 h. Serially diluted IgGs (Biolegend, San Diego, CA, USA) were used as positive controls with isotype IgGs serving as negative controls. HRP-labeled anti-human IgG for minibody detection or HRP-labeled anti-mouse IgG (BioRad, Hercules, CA, USA) for anti-human antibody detection were added and incubated at room temperature for 1hr and then developed. Absorbance was measured using Tecan plate reader (TECAN, Männedorf, Switzerland).

### 2.7. Flow Cytometry

The following monoclonal antibodies conjugated with fluorochrome were used: Desmoglein-2(Santa Cruz Biotechnology), 41BBL, ICOSL (BioLegend), OX40L, CAR, CD46 (BD Biosciences), FAP (R&D Systems). Cells were stained with these antibodies for 30 min at 4 °C. Live/dead discrimination was determined via inclusion of 7-aminoactinomycin D (7AAD; BD Pharmingen). Stained cells were analyzed using Gallios flow cytometer with Kaluza software (BD Biosciences) according to the manufacturer’s instructions.

### 2.8. Co-Culture Experiments

*ffLuc*-expressing target cells were seeded in 48-well plates. After 24 h, A549 supernatant infected with HDAds described in the figure legends were added to the target cells with effector cells at the effector to target ratio denoted in the figure legends. Residual live target cells (*ffluc* activity) were measured using a Luciferase Assay System (Promega, Madison, WI, USA) and measured by plate reader (BMG Labtech, Orlenberg, Germany) at the timepoints described in the figure legends.

### 2.9. RNA Extraction and RT-PCR

SUM-159 cells were harvested and RNA was extracted using RNeasy Plus Mini kit (Qiagen, Hilden, Germany). RNA samples were converted to cDNA using Super Script III First-Strand Synthesis System (Thermo Fisher, Waltham, MA, USA). The levels of human cancer/testis antigen were quantified using CFX96 Real-Time PCR Detection System (BioRad) and normalized with human B-Actin. We obtained all primer sets from Bio-Rad.

### 2.10. MTS Assay

Ten thousand cells per well were seeded into 96-well plates. Cells were infected with OAd, HDAd, or CAd (OAd:HDAd = 1:1) at the doses indicated in the figure legends and incubated at 37 °C for 96hrs. Cell viability was analyzed by MTS assay according to the manufacturer’s instruction (Promega). Cell viability was normalized to that of untreated cells.

### 2.11. Statistics and Reproducibility

Results are represented of two or more independent experiments. Data with three or more groups were analyzed by ordinary one-way ANOVA analysis. Data were analyzed with GraphPad Prism 9 (Dotmatics, Boston, MA, USA).

## 3. Results

### 3.1. Switching Ad Serotypes Enables Targeting of a Wide Range of Solid Tumor Cell Lines

In addition to their large transgene capacity, the ease with which HDAd capsids can be modified is another advantage. Ad transduction is mediated by the interaction of the Ad fiber knob domain with its cognate cellular receptor [22]. In the case of group C Ads, the most well studied, Ad5 uses the coxsackie and adenovirus receptor (CAR) as its entry receptor. Though CAR is expressed on most cells of epithelial origin, it is located on their basolateral surface, making it less accessible due to tight junctions between cells. The receptor for group B1 Ads, including Ad35, is CD46, which is ubiquitously expressed on human cells (except erythrocytes) and is often upregulated on malignant cells [23,24,25]. Group B2 Ads, such as Ad3, utilize desmoglein-2 (DSG-2) as their primary receptor [26], which can be more accessible than CAR even though it is a cellular junction molecule, as it resides more on the apical side. These Ads, including Ad3, can also use CD46 when it is expressed at high levels, such as on malignant cells [27].

**Figure 1:**
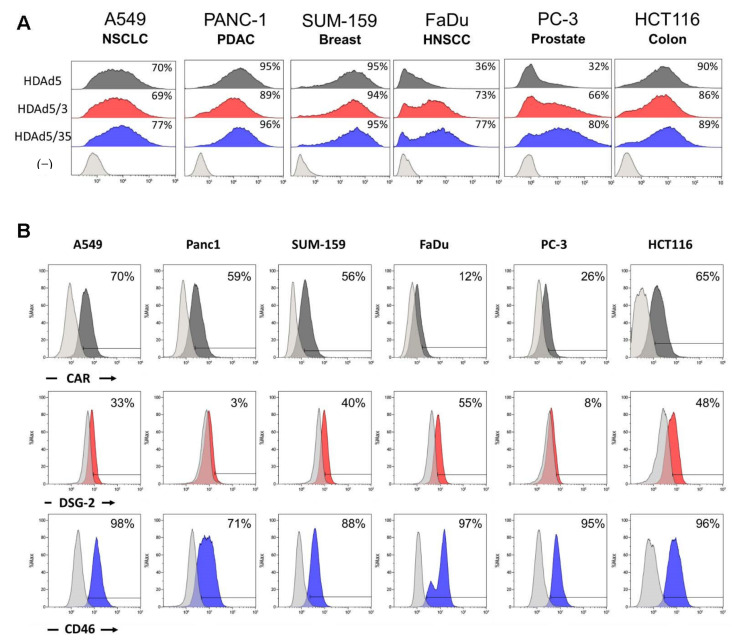
Adenovirus knob chimeras mediate differential tumor type tropism. (**A**) A panel of solid tumor cell lines (A549, Panc-1, SUM-159, FaDu, PC-3, HCT116) were infected with 100 vp/cell of HD5*eGFP,* HD5/3*eGFP* or HD5/35*eGFP* for 1 h. GFP expression was assessed via flow cytometry 24 h post-infection. (**B**) Cell surface expression of Ad receptors on the panel of solid tumor cell lines were assessed via flow cytometry.

As their name suggests, HDAds rely on a helper virus (HV) during vector production to provide all of the capsid proteins in trans to form complete viral particles. Once an HDAd is generated there is no need to make additional genome modifications to alter the capsid, one can simply utilize a HV with the desired capsid during the manufacture process [14]. In this way, HDAds with identical expression cassettes can have altered tropism in a wide range of cell types. Here, we used a panel of various solid tumor cell lines to evaluate the transduction efficacy of HDAd5, and fiber-knob chimeras HDAd5/3 and HDAd5/35, expressing the same GFP reporter expression cassette (Figure 1A and Figure S1). 

We found the three serotypes had similar transduction efficacy in most cancer cell lines. HDAd5 had lower transduction in FaDu (head and neck squamous cell carcinoma) and PC-3 (prostate adenocarcinoma). CAR is expressed at lower levels in these lines correlating to their reduced transduction by HDAd5 (Figure 1B). The HDAd5/3 vector and HDAd5/35 vector transduced most cell lines equally well, with the exception of PC-3, which was only transduced by HDAd5/3 at an intermediate level. Although DSG-2 expression is lower than CD46 in all lines, the Ad3 knob can utilize both DSG2 and CD46 for infection [27], leading to similar transduction as HDAd5/35 capsid vectors. Since the Ad5/3 chimeric capsid has confirmed safety in numerous OAd clinical trials [28,29,30], we selected the HDAd5/3 chimeric capsid to evaluate the function of our HydrAd system. However, as mentioned above, if necessary we can easily switch HDAd capsid components using the appropriate HV during production as well as the OAd partner within the CAdVEC platform. 

### 3.2. HDAd-Mediated Expression of Immunomodulatory Molecules

Due to the intrinsic immunogenicity of Ad vectors, they are commonly delivered directly into the tumor mass. This route requires lower doses than systemic infusion, as the total dose is delivered directly to the target, avoids encountering pre-exisiting neutralizing antibodies in the blood, and importantly, leads to expression of therapeutic transgenes within the treated tumor microenvironment (TME) [31,32], ensuring that the transgenes are expressed where they can be most effective and minimizing associated toxicities. To evaluate the feasibility of generating HDAds expressing therapeutic transgenes to modulate various tumor suppressive pathways, we created a library of expression cassettes belonging to six functional categories (Table 1).

#### 3.2.1. Chemokines

**Figure 2:**
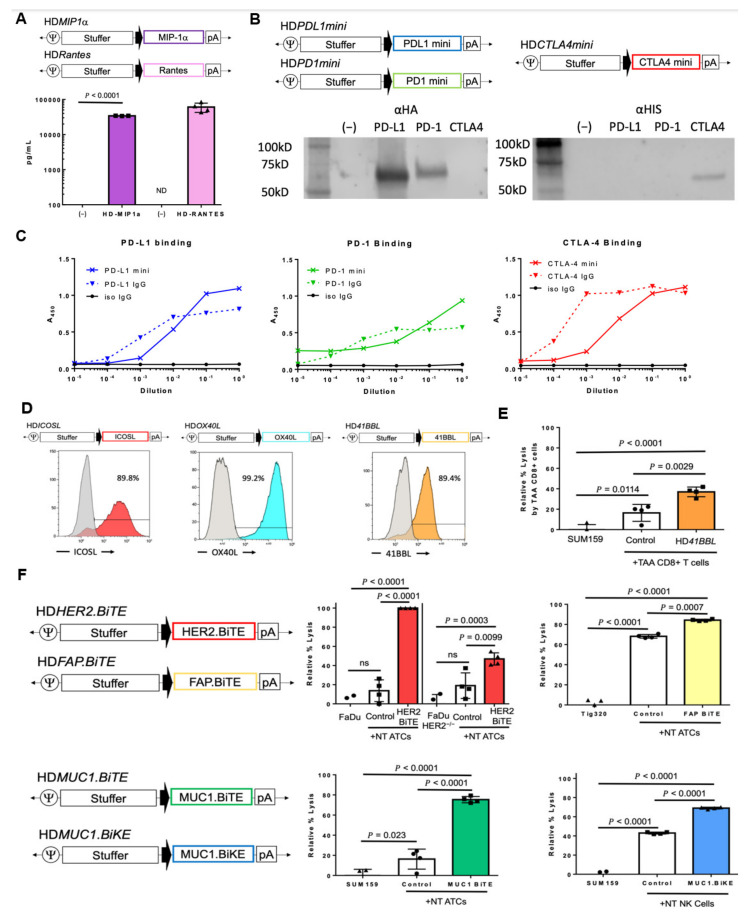
Expression and function of HDAd encoded immunomodulatory molecules. (**A**) Schematic structure of HD*MIP1α* and HD*Rantes*. PC-3 cells were infected with 1000 vp/cell HD5/3*MIP1α* or control HDAd [(−)](*n* = 3), A549 cells were infected with 1000 vp/cell HD5/3*Rantes* or control HDAd [(−)] (*n* = 4). PC-3 media were collected 48 h, A549 at 24 h post-infection, and chemokine levels in the media were measured by ELISA. Data are presented as means ± SD. ND = not detectable. (**B**) Schematic structure of HD5/3*PDL1mini,* HD5/3*PD1mini,* and HD5/3*CTLA4mini*. A549 cells were infected with 100 vp/cell HDAds expressing minibody or control HDAd [(−)], media were collected 48 h post-infection and subjected to western blotting for PDL1 minibody, PD1 minibody, which was detected by anti-HA antibody, and CTLA4 minibody which was detected by anti-HIS antibody. Uncropped blots of Figure 2B are shown in Figure S2 (**C**) Media containing minibody was also assessed for binding to recombinant human protein (PDL1mini:PDL1, PD1mini:PD1, CTLA4mini:CTLA4) by ELISA. Respective IgGs or Isotype IgG were used as controls (10 ug/mL; highest concentration). (**D**) Schematic structure of HD5/3*ICOSL*, HD5/3*OX40L*, and HD5/3*41BBL*. A549 cells were infected with 1000 vp/cell HD5/3*ICOSL*, HD5/3*OX40L*, or HD5/3*41BBL*. Cell surface expression of the respective co-stimulatory ligand expression was assessed by flow cytometry 48hrs post-infection. (**E**) SUM-159 expressing *ffLuc* cells were infected with 100 vp/cell HD5/3*41BBL*. Tumor associated antigen-specific (TAA) CD8+ T-cells were added 24 h post-infection (effector to target ratio, E:T = 1:1). Cells were harvested 96 h post-co-culture, and viable cancer cells were analyzed by luciferase assay. Data are presented as means ± SD (*n* = 4). (**F**) Schematic structure of HD5/3*HER2.BiTE*, HD5/3*FAP.BiTE*, HD5/3*MUC1.BiTE,* and HD5/3*MUC1.BiKE*. A549 cells were infected with 100 vp/cell HDAds expressing BiTE, BiKE or control HDAd [(-)], media were collected 48 h post-infection. Supernatant containing HER2.BiTE, MUC1.BiTE, or MUC1.BiKE were applied to cancer cells (FaDu, SUM-159) or supernatant containing FAP.BiTE were applied to Tig-3-20 cells expressing *ffLuc* in the presence of non-transduced T or NK cells (E:T = 1:10). Cancer cells were harvested 72 h post-co-culture, Tig-3-20 cells were harvested 48 h post-co-culture, and viable cells were analyzed by luciferase assay. Data are presented as means ± SD (*n* = 4), ns-non significant.

To attract functional cytotoxic immune cells to the tumor site, we generated HDAds expressing chemokines that induce immune infiltration into the tumor mass. Macrophage inflammatory protein MIP1*α* (CCL3) has broad chemotactic function for T and NK cells [33] and RANTES (CCL5) is a chemoattractant for T cells [34]. An OAd expressing RANTES enhanced adoptively transferred chimeric antigen receptor -modified T cell (CAR-T) infiltration at the tumor site in preclinical studies [35]. We showed that cancer cells infected with HDAds encoding MIP1*α* or Rantes specifically expressed approximately 34,000 pg/mL and 61,000 pg/mL of chemokine, respectively (Figure 2A). As different chemokines recruit different immune cells through their chemokine receptors, we could add chemokine(s) into our HDAd to recruit specific immune cell(s) to the tumor site as needed (e.g., immunological “cold” tumor type), based on results from our ongoing clinical trial.

#### 3.2.2. Cytokines

Once effector immune cells are brought to the tumor site by chemokines, they must become fully activated. T cells require three signals for complete triggering of their cytotoxic functions: (i) target recognition, (ii) proliferation, and (iii) persistence. Proliferation is dependent on cytokine support. Intravenous administration of recombinant cytokines is associated with clinical toxicities [36], however, providing cytokine support via a gene therapy vector at the tumor site creates a gradient of expression with the highest concentration at the tumor site and lower amounts present in circulation. Thus, this approach should enhance local activation and proliferation of immune cells with less risk of systemic toxicity. We previously demonstrated that our HDAds express their encoded cytokine (listed in Table 1), and that these cytokines are functional and elicit phosphorylated signal transducers and activators of transcription (STATs) in CAR-T cells, leading to long-term persistence of CAR-T cells in animal models [11]. Since these cytokines activate different STAT pathways and different immune populations, in future studies we can co-express different cytokines in our vector.

#### 3.2.3. Immune Checkpoint Inhibitors

Immune checkpoint antibodies (ICIs) are some of the most promising cancer therapeutics to be clinically approved in recent years. One mechanism of tumor immunosuppression is the upregulation of inhibitory ligands on tumor cells, which bind their cognate receptors on T cells, repressing activation (acting as a “brake”) [37]. The most famous of these immune checkpoint interactions are CD80/CD86-CTLA4 and PDL1-PD1. Antibodies against CTLA4, PDL1, and PD1 have been approved for clinical use, however single ICIs are often insufficient to eradicate tumors [38,39]. Although combining ICIs targeting different checkpoint pathways improves clinical outcomes and is approved for some tumor types, systemic treatment of multiple ICIs causes severe toxicities [40]. We have previously reported an HDAd expressing a PDL1 minibody and demonstrated its superior activity and safety when HD*αPDL1mini* as CAdVEC is delivered intratumorally compared to systemic administration of a PDL1 IgG in combination with adoptively transferred CAR-T cells in xenograft mouse models [10]. In addition to HDa*PDL1mini,* we now generated *α*PD1-minibody and *α*CTLA4-minibody expression cassettes (Figure 2B). These ICIs bind specifically to their cognate proteins similarly to or, in the case of CTLA4, better than commercially available IgGs (Figure 2C, Figure S3). Our minibodies dimerize after expression [10], suggesting that if we co-express different minibodies (e.g., *α*PD-1 and *α*CTLA-4), they may form hetero-dimers and act as bi-functional minibodies at tumor sites. 

#### 3.2.4. T-Cell Co-Stimulatory Molecules

In addition to antigen stimulation (signal 1) and cytokine proliferation (signal 3), T cells require a co-stimulatory signal (signal 2) for complete activation. Although providing ICIs can “release the brake” and remove inhibitory signaling, a co-stimulation signal is still required. Co-stimulatory ligands are often lacking on tumor cells, but are instead typically expressed on professional antigen presenting cells (APCs) that have limited capacity for tumor infiltration [41]. It stands to reason then that forced expression of T cell co-stimulatory molecules at the tumor site could help fully activate endogenous tumor-infiltrating T cells. We constructed HDAds encoding either inducible T cell costimulator ligand (ICOSL), OX40L, or 41BBL, for which agonistic antibodies have been tested in clinical trials [42,43], and confirmed cancer cell surface expression of these molecules after infection (Figure 2D). To evaluate the proof-of-principle that HDAd-derived co-stimulatory molecules enhance the function of tumor associated antigen-specific T cells (TAA-T cells), we tested the ability of HD*41BBL* to enhance the cytotoxicity of TAA-T cells, as 41BB is expressed on CD8+ T cells after activation [44]. We co-cultured tumor associated antigen (TAAs: SSX2, PRAME, MAGEA4, SURVIVIN, and NY-ESO-1) specific CD8+ T cells with partially HLA-matched cancer cells expressing SURVIVIN, MAGE-A3, and MAGE-A4 (Figure S4). SUM-159 breast cancer cells infected with HD*41BBL* were killed two-fold more efficiently by TAA-T cells compared with cells infected with an HDAd containing no transgene (HDAd0) (Figure 2E). These results indicate that HDAd-derived co-stimulatory ligand expression on cancer cells provides “signal 2” to tumor-specific T cells to enhance their function.

#### 3.2.5. Engager Molecules

As mentioned above, T cells require antigen specific recognition for engagement with a cellular target for activation. Solid tumors often evade immune recognition by downregulating antigen presentation machinery leaving immune cells with no immunogenic target [45]. Bi-specific engager molecules are adapter proteins that allow any T cell (BiTE) or NK cell (BiKE) to interact with tumor cells expressing the cognate molecule on their surface. CD44 variant 6 (CD44v6) has tumor-restricted expression, making it a good candidate for redirected T cell cytotoxicity [46]. We demonstrated additive anti-tumor activity by providing HD*CD44v6.BiTE* to CAR-T cells for dual antigen targeting and the ability to redirect HER2-specific CAR-T cells to kill HER2-negative tumor cells through CD44v6 engager, in contrast to a control HD*CD19.BiTE* even in an orthotopic (metastatic) mouse model [12]. To expand potential target cells for engager molecules using our gene therapy approach, we developed three additional BiTEs and one BiKE (Table 1).

We reported the efficacy of targeting a variety of solid tumor types expressing different HER2 levels with HER2.CAR-T cells [10,11,12,13]. Unlike a targeting antibody, which relies on antibody-dependent cellular cytotoxicity (ADCC), our HER2.CAR-T cells can recognize and kill cancer cells with low HER2 expression [47]. Here, we tested whether the same HER2.scFv-derived HER2.BiTE provides unmodified T cells with a mechanism for antigen targeting. Supernatant containing HER2.BiTE induced efficient killing (>90% lysis) of HER2+ FaDu cells (Figure S4) by non-transduced (NT) T cells that could not kill HER2-knock out FaDu cells as efficiently (Figure 2F). 

Destruction of tumor stromal components is another mechanism by which immunotherapies can overcome the complex immunosuppressive structure of the TME [48]. Fibroblast activating protein (FAP) is expressed on cancer associated fibroblasts (CAFs), which promote cancer invasion and tumorigenic angiogenesis [49]. To target CAFs, we generated an HD*FAP.BiTE* vector that increased NT cell-mediated lysis of FAP+ CAF-like cells (Figure S4) to 84% compared to 68% lysis in the absence of the BiTE (Figure 2F).

Glycosylated proteins, such as Mucin1 (MUC1), are being investigated as cancer-specific targets as they can have abberant glycosylation patterns and are overexpressed in some cancers [50]. Supernantant containing a MUC1.BiTE enhanced unmodified T cell killing of MUC1+ triple negative breast cancer cell line SUM-159 (Figure S4) from 16% to 75% (Figure 2F). 

Our recent work suggests that CAdVEC activates endogenous NK cell anti-tumor activity in humanized mouse models [13]. To enhance NK cell tumor specificity, we generated a MUC1.BiKE to provide antigen engagement to these NK cells, and confirmed that the MUC1.BiKE increased NK cell killing of SUM-159 cells to 69% lysis compared to 43% at baseline (Figure 2F). HDAds are a customizable platform to express BiTE and/or BiKE molecules for specific tumor antigen targeting by endogenous or adoptively transferred effector cells, expanding their cytotoxic activity to multiple antigenic targets.

### 3.3. In-Situ Amplification of HDAd-Encoded Transgenes with Viral-Mediated Oncolysis

**Figure 3:**
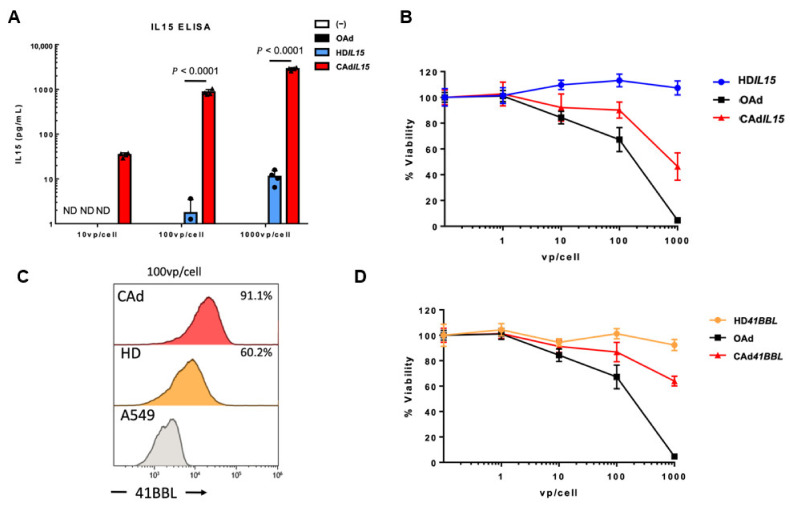
Co-infection with OAd amplifies HDAd encoded transgenes and maintains oncolysis. (**A**) A549 cells were infected with a total of 10 vp/cell, 100 vp/cell, or 1000 vp/cell of HD5/3*IL15* or CAd5/3*IL15* (OAd:HDAd, 1:1). Medium samples were collected 24 h post-infection. The levels of IL15 in medium samples were quantified by IL15 ELISA assay. Data are presented as means ± SD (*n* = 4). ND = Not detectable. (**B**) A549 cells were infected with increasing doses of HD5/3*IL15* or OAd or with CAd5/3IL15 (OAd:HDAd, 1:1). Viable cells were analyzed at 96 h by MTS assay. Data are presented as means ± SD (*n* = 6). (**C**) A549 cells were infected with a total of 100 vp/cell of HD5/3*41BBL* or CAd5/3*41BBL* (OAd:HDAd, 1:1). Cell surface expression of 41BBL expression was assessed by flow cytometry 48 h post-infection. (**D**) A549 cells were infected with increasing doses of HD5/3*41BBL* or OAd5/3 or CAd5/3*41BBL* (OAd:HDAd, 1:1). Viable cells were analyzed at 96hrs by MTS assay. Data are presented as means ± SD (*n* = 6).

As mentioned above, our CAdVEC system has the added benefit of HDAd-encoded transgene amplification in co-infected cells through replication of both the OAd and HDAd [9]. Thus, infection with CAdVEC leads to higher levels of HDAd-derived transgene expression than HDAd infection alone. In our previously reported HDAd cytokine library, we achieved a high level of cytokine expression from all vectors except HD*IL15* [11]. Production of IL15 is tightly regulated and exogenous expression is notoriously difficult [51]. As demonstrated here, IL15 was undetectable in A549 cells infected with 10 vp/cell of HD*IL15*, however, when cells were coinfected with OAd (CAdVEC) 10 vp/cell total, we detected 34 pg/mL of IL15 (Figure 3A). This effect is dose dependent, as we were able to further enhance IL15 expression at doses of 100 vp/cell and 1000 vp/cell. Since CAdVEC contains a 1:1 ratio of HDAd:OAd, this system provides both HDAd-derived transgene amplification and direct lytic effects from the oncolytic virus (Figure 3B).

A similar effect is achieved with coinfection of an HDAd encoding a co-stimulatory molecule. As demonstrated in Figure 2D, 1000 vp/cell of HD*41BBL* is required for 90% expression efficacy. Using CAdVEC, we produced the same level of 41BBL expression with only 100 vp/cell (Figure 3C), and we observed dose-dependent lytic effects similar to those of CAdVEC with HD*IL15* (Figure 3B,D). These data demonstrate the significant advantage of CAdVEC in enhancing HDAd-mediated transgene expression at lower viral doses. 

### 3.4. Complex Transgene Constructs

#### 3.4.1. HDTrio

**Figure 4:**
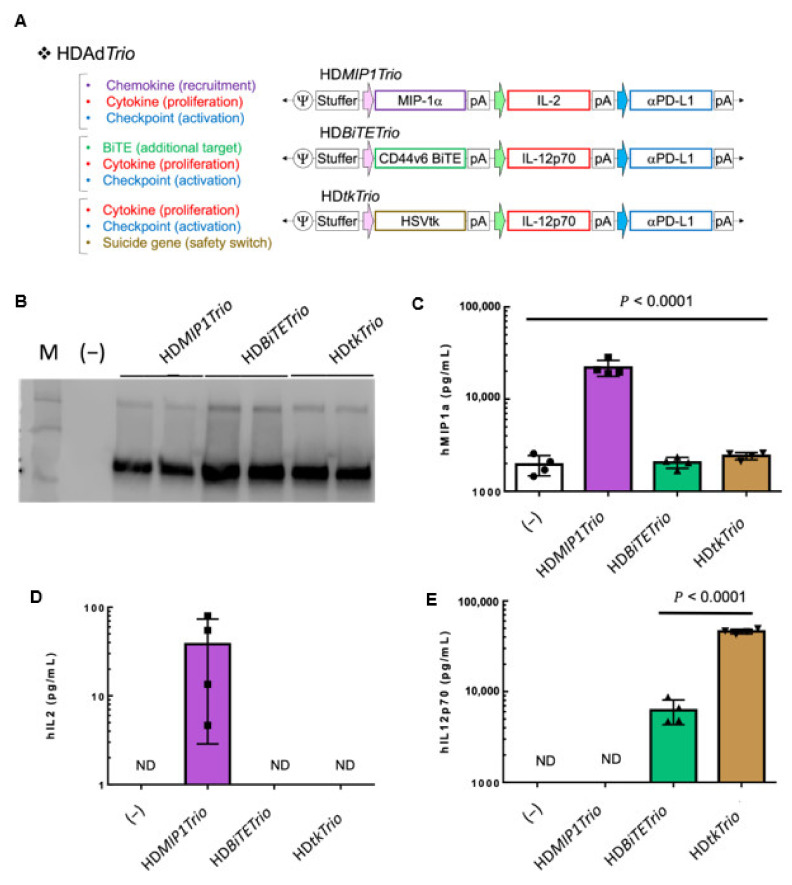
Simultaneous expression of different transgenes from HDTrio. (**A**) Schematic structure of HD5/3*Trios*. HD5/3*MIP1Trio* encodes human MIP1*α*, IL2, and anti-PDL1 minibody expression cassettes. HD5/3*BiTETrio* encodes the CD44v6.BiTE, human IL12p70, and anti-PDL1 minibody expression cassettes. HD5/3*tkTrio* encodes the Herpes simplex virus thymidine kinase (HSVtk) safety switch, human IL12p70, and anti-PDL1 minibody expression cassettes. (**B**) A549 cells were infected with 100 vp/cell of HD5/3*MIP1Trio*, HD5/3*BiTETrio*, or HD5/3*tkTrio*. Media were collected 48 h post-infection. Medium samples were subjected to western blotting for PDL1 minibody, which is detected by anti-HA antibody. Uncropped blot of Figure 4B is shown in Figure S5. (**C**) Medium samples were analyzed for human MIP1*α*, (**D**) human IL2, and (**E**) human IL12p70 by ELISA assay. Data are presented as means ± SD (*n* = 4). ND = Not detectable.

Thus far, we have demonstrated the diversity of immunomodulatory transgenes we can produce using an HDAd vector system. However, the unique benefit of the HDAd is that the 34 kb transgene capacity affords the ability for multiple transgene expression cassettes in a single vector. As discussed above, a multi-pronged approach targeting multiple tumor immunosuppressive pathways simultaneously will be required to successfully eradicate advanced tumors. Therefore, we have used HDAd vectors in a mix-and-match approach for multiple immune pathway activation; we now call this vector platform the HydrAd. Presented here are what we refer to as HD*Trio*: HDAds encoding three transgene expression cassettes (Figure 4A). We have previously reported the function of CD44v6 [12] and HSVtk HD*Trio*s [13]. The HD*tkTrio* is currently being evaluated in a clinical trial as part of our CAdVEC system in combination with HER2.CAR-T cells at Baylor College of Medicine (ClinicalTrials.gov Identifier: NCT03740256). 

Here we present the HD*MIP1Trio* and evaluate the transgene expression of other *HDTrios* side-by-side. The three HD*Trios* encode the HA-tagged aPDL1 minibody in which the binding function was confirmed in Figure 2C. Even when the aPDL1 minibody is encoded with two other transgenes it is still efficiently expressed (Figure 4B). MIP1a and IL2 are selectively expressed, 22,000 pg/mL and 38 pg/mL respectively, from HD*MIP1Trio* (Figure 4C,D). The IL12p70 expression is also confirmed from HD*BiTETrio* (6000 pg/mL) and HD*tkTrio* (46,000 pg/mL) (Figure 4E). These results indicate that three different transgene expression cassettes in a single HDAd vector express functional molecules regardless of their sequences and promoters.

#### 3.4.2. HDTetra

**Figure 5:**
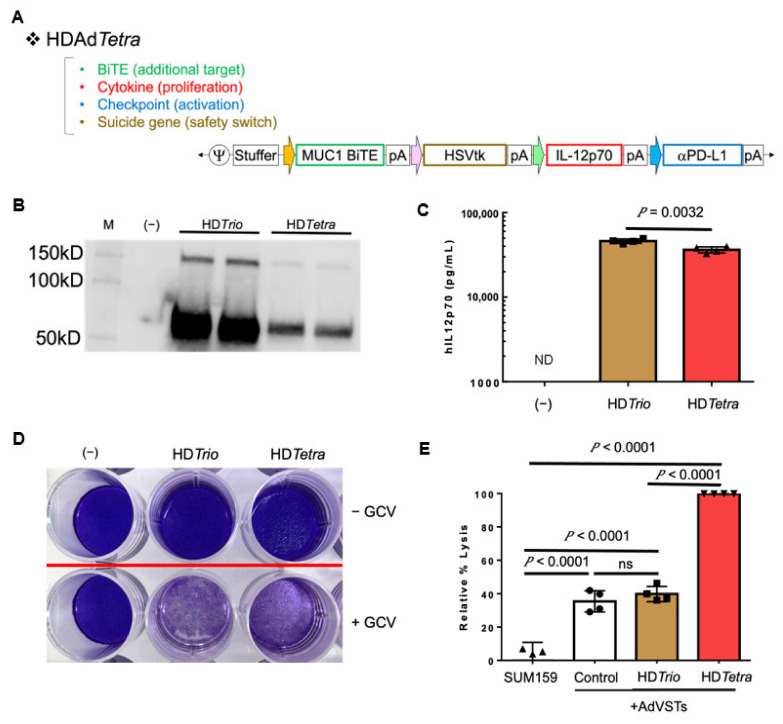
HDTetra enables simultaneous expression of four transgenes. (**A**) Schematic structure of HD5/3*Tetra* encoding the MUC1.BiTE, HSVtk safety switch, human IL12p70, and anti-PDL1 minibody expression cassettes. (**B**) A549 cells were infected with 500 vp/cell of HD5/3*tkTrio* (HD5/3*Trio*) or HD5/3*Tetra*. Media were collected 48hrs post-infection. Medium samples were subjected to western blotting for PDL1 minibody, which is detected by anti-HA antibody. Uncropped blot of Figure 5B is shown in Figure S6 (**C**) A549 cells were infected with 100 vp/cell of HD5/3*Trio* or HD5/3*Tetra*. Media were collected 48 h post-infection. Medium samples were assessed for human IL12p70 by ELISA assay. Data are presented as means ± SD (*n* = 4). ND = Not detectable. (**D**) A549 cells were infected with 500 vp/cell of HD5/3*Trio* or HD5/3*Tetra*. Media containing ganciclovir (GCV) was added to denoted wells 24 h post-infection and replaced daily. After 8 days viable cells were fixed and stained with crystal violet. Representative image presented. (**E**) A549 cells were infected with 500 vp/cell of HD5/3*Trio* or HD5/3*Tetra*, supernatants were collected 48 h post-infection. Supernatant containing MUC1.BiTE were applied to SUM-159 cells expressing *ffLuc* in the presence of adenovirus-specific T-cells (AdVSTs) (E:T = 1:5). Cancer cells were harvested 72 h post-co-culture and viable cells were analyzed by luciferase assay. Data are presented as means ± SD (*n* = 4), ns-non significant.

We have demonstrated that incorporating additional transgenes into the HDAd can stimulate multiple immune pathways for additive effects [11,12,13]. HD*tkTrio* is currently being evaluated as part of our CAdVEC system in a Phase I trial (NCT03740256), and we have not observed any adverse event in patients. OAd trials have shown the development of Ad-specific T cells (AdVSTs) in patients [6,28], and we also observed AdVST development in patients treated with CAdVEC. We expect that these AdVSTs infiltrate Ad-treated tumor sites and eliminate Ad-infected cells, possibly reducing the lytic effects of OAd. We hypothesize that engaging these AdVSTs (“irrelevant” T cells) with cancer cells will enhance the antitumor effects of CAdVEC. To test this hypothesis, we incorporated the MUC1.BiTE into our clinical HD*tkTrio,* creating HD*Tetra* (Figure 5A). We successfully rescued HD*Tetra* and confirmed expression of the αPDL1 minibody (Figure 5B) and IL12p70 cytokine (Figure 5C). Although HD*Tetra* has slightly reduced transgene expression compared to HD*tkTrio*, this can be overcome by co-infection with OAd (CAdVEC system), as we demonstrated in Figure 3. Even with reduced expression, the transgenes remain functional, as demonstrated by the ganciclovir-dependent killing of HD*Tetra* infected cells (Figure 5D). We further found that supernatant containing the MUC1.BiTE from HD*Tetra* infected cells enabled AdVSTs to effectively kill MUC1+ cells in vitro (Figure 5E), confirming the potential of this engager to re-target “irrelevant” effector cells against cancer cells. These results indicate that we can make additional modifications to our HDAd for the next generation of CAdVEC. We will use clinical samples obtained from our ongoing trial to address which additional immunomodulation(s) are required for CAdVEC to be most effective in the future.

## 4. Discussion

A multi-targeted cancer immunotherapy approach is needed to combat the complex solid tumor microenvironment. Cancer gene therapy has enormous potential to provide therapeutic transgenes directly within the TME and using a large capacity vector enables delivery of multiple transgenes to target multiple suppressive pathways simultaneously.

**Figure 6:**
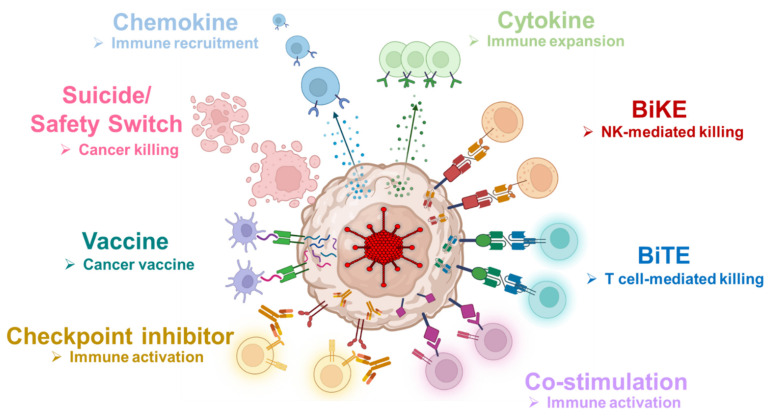
Concept of the HydrAd platform. The large transgene capacity of the HDAd enables the simultaneous expression of multiple immunomodulatory transgenes. The HydrAd platform can be adapted and customized as needed to overcome the specific challenges of various solid tumor types.

Adenoviral gene therapy vectors have proven their efficacy over the past decades, most recently as COVID-19 vaccine vectors [52,53], and their safety in gene and cancer therapy applications [54]. HDAd vectors provide an ideal platform for cancer gene therapy because they have broad tumor tropism, similar to other Ad vectors, and can encode multiple transgene expression cassettes to facilitate activation of diverse immunomodulatory signaling pathways (Figure 6). Here, we created a library of HDAds encoding single transgenes from six classes of immunostimulatory molecules (Table 1) and demonstrated their function and capacity for effector cell activation.

We capitalized on the easy manipulation of the Ad genome to generate an OAd that not only specifically lyses cancer cells, but when co-infected with our HDAd vectors in the CAdVEC system, provides helper function and simultaneous HDAd replication, amplifying expression of transgenes derived from HDAd while maintaining lytic function of OAd [9,10,11,12,13]. 

We know that solid tumors utilize numerous immunosuppressive pathways to escape immune surveillance, so a single immunomodulatory molecule is unlikely to overcome all of these inhibitory mechanisms [7,8]. We previously demonstrated that incorporating additional therapeutic transgenes into HDAd has an additive impact in our CAdVEC, allowing simultaneous targeting of cancer cells and elements of the suppressive microenvironment. We have termed this HDAd platform the HydrAd for its potential for multi-pronged mechanisms to stimulate immune effector cells to eradicate tumors (Figure 6). For instance, here we describe an HD*Tetra* HydrAd that expands our clinical HD-HSVtk-IL12p70-aPDL1 “Trio” by incorporating an engager molecule for T cell retargeting.

The HydrAd platform enables us to combine therapeutic transgenes to attack the suppressive profile of specific tumors. For example, immunologically cold tumors (e.g., pancreatic cancer) [55] may require chemokines to bring effector cells to tumor targets, an engager molecule to re-direct “irrelevant” effectors (e.g., virus-specific T cells) to malignant cells, and immune checkpoint blockade with co-stimulation to fully activate immune cells. In a “hot” tumor with immune cells present (e.g., lung cancer) [56], the HydrAd could be used in an in situ vaccine approach, expressing tumor antigens and cytokines to activate professional antigen presenting cells as well as effector T cells with immune checkpoint blockade to release their full cytolytic potential. Since each tumor type has a unique microenvironment, we will optimize the immunomodulatory molecules encoded in our HDAd to maximize anti-tumor immunity [13]. 

It is generally accepted that while direct lysis is an important component of oncolytic viral (OV) therapy, immunostimulation is crucial for development of widespread anti-tumor effects and immunological memory through local OV treatment. Further exploration of the proportional contribution of OAd-mediated oncolysis versus the immunostimulation provided by the HDAd-encoded transgenes to anti-tumor immunity is warranted. We have begun to explore this further in our humanized mouse models [13], and will study the effect of our treatment in patients as part of our ongoing clinical trial (NCT03740256). HydrAd has the potential to synergize not only with endogenous immune cells but also with adoptively transferred immune cells, such as CAR-modified cells. We reported promising results from studies with HD*Trios* as part of our CAdVEC system when administered with HER2.CAR-T cells in different animal models, including orthotopic and humanized mouse models [12,13]. In humanized mice, CAd-*tkTrio* enhanced the function of the adoptively transferred CAR-T cells and endogenous immune responses. 

The inherent immunomodulation of OAds and the engineered stimulation provided by the HydrAd platform has potential to synergize with numerous treatment modalities. Many clinical trials have explored combining oncolytic virotherapy with standard of care chemo- and radiotherapies, as they provide tumor debulking. While combining effective treatments has some promise, a careful balance must be struck as induction of immune responses against the tumor is a crucial mechanism of action for OVs and chemotherapies could hinder the robustness of cytotoxic immune cells. To that end, numerous trials are evaluating the combination of OVs with immune checkpoint inhibitors to take advantage of the immunostimulation provided by OVs [57].

We are currently investigating the promising combination of CAd-*tk-Trio* with adoptively transferred HER2.CAR-T cells in a first-in-human clinical trial for advanced HER2+ solid tumors at Baylor College of Medicine (NCT03740256). Based on results from these studies, we will take advantage of the flexibility of the HydrAd platform to modify our current clinical HD*Trio* to further enhance CAdVEC anti-tumor effects alone and in combination with adoptive cell therapies.

## 5. Conclusions

Successful cancer immunotherapy will require simultaneous modulation of multiple inhibitory pathways presented by solid tumors. We have generated a library of HDAd vectors expressing various classes of immunomodulatory molecules with demonstrated functionality in multiple solid tumor cell lines. We also validated our previously established combinatorial approach using an oncolytic adenoviral vector to amplify low-expressing transgenes encoded within a co-infected HDAd.

As the major benefit of using HDAds is the large transgene capacity, we have developed a platform, the HydrAd, to simultaneously express multiple transgenes from a single HDAd vector. Thus, enabling one HydrAd to target multifaceted immunosuppressive mechanisms presented by solid tumors. We confirmed the expression and function of transgenes expressed from HDAds encoding multiple transgene cassettes targeting different immune pathways. The HydrAd presents a promising cancer gene therapy strategy to target complex solid tumors.

## Figures and Tables

**Table 1:** Library of HDAds encoding immunomodulatory molecules.

Chemokine	Cytokine	Checkpoint Inhibitor	Co-Stimulation	Engager	Suicide Gene
MIP-1α	IL-2	αPD-L1 mini-body	4-1BB ligand	CD19 BiTE	HSTtk
RANTES	IL-7	αPD-1 mini-body	OX40 ligand	CD44v6 BiTE	
	IL-12p70	αCTLA-4 mini-body	ICOS ligand	MUC-1 BiTE	
	IL-15			FAP BiTE	
	IL-21			HER2 BiTE	
				MUC-1 BiKE	

## Data Availability

Data presented I this study are available in the present article and supplementary materials.

## Supplementary Materials

The following supporting information can be downloaded at: www.mdpi.com/article/10.3390/cancers14112769/s1, Figure S1: HD*eGFP* transduction microscopy; Figure S2: Complete, uncropped western blots for Figure 2; Figure S3: Minibody binding controls; Figure S4: Tumor target antigen expression; Figure S5: Complete, uncropped western blots for Figure 4; Figure S6: Complete, uncropped western blots for Figure 5.
