# Supplementary material for: HydrAd: A Helper-Dependent Adenovirus Targeting Multiple Immune Pathways for Cancer Immunotherapy
Source: Cancers (Basel). 2022 Jun 2;14(11):2769. doi: 10.3390/cancers14112769 (PMC9179443; doi:10.3390/cancers14112769)
Supplement: Supplementary file 1 [file cancers-14-02769-s001.zip › cancers-1724688-supplementary.pdf]

cancers MDPI

Supplementary Materials: HydrAd: A Helper-Dependent Adenovirus Targeting Multiple Immune Pathways for Cancer Immunotherapy

Amanda Rosewell Shaw, Caroline Porter, Greyson Biegert, Lisa Jatta and Masataka Suzuki

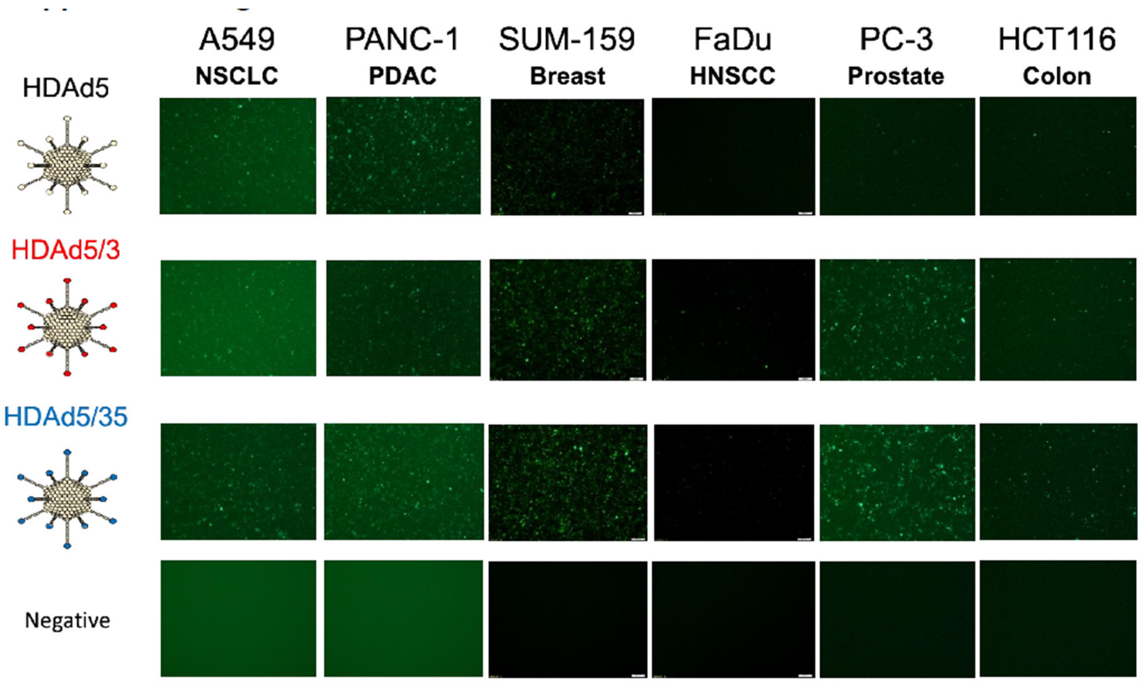

Figure S1. HDeGFP Transduction Microscopy. Representative fluorescent microscopy images of HD*eGFP* 100vp/cell infected solid tumor cell line panel.

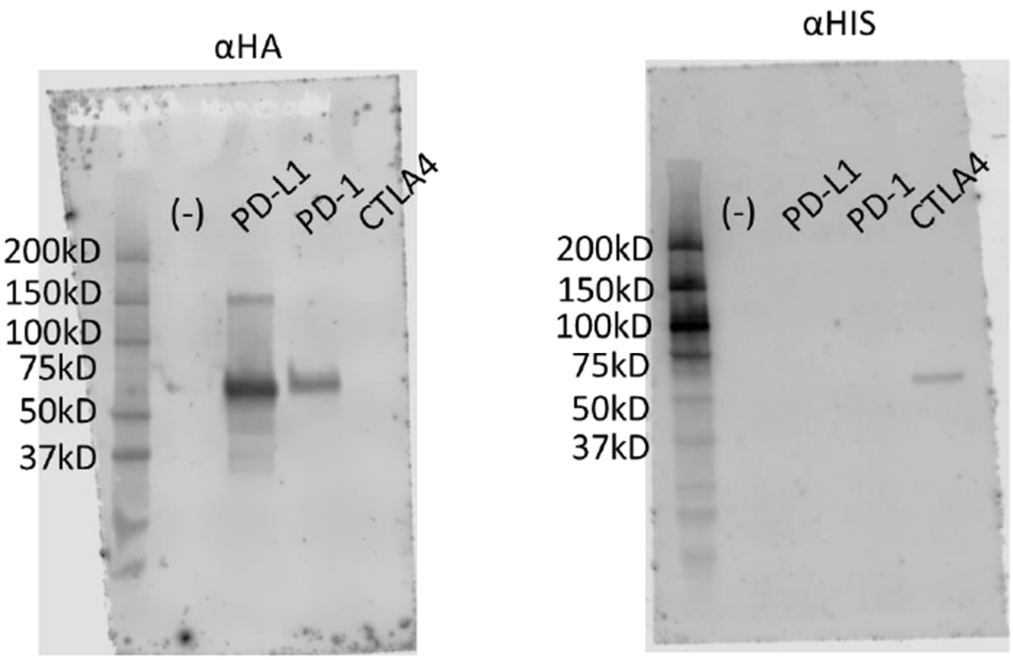

Figure S2. Complete, uncropped western blots for HD*PDL1*, HD*PD1*, HD*CTLA4* minibody expression.

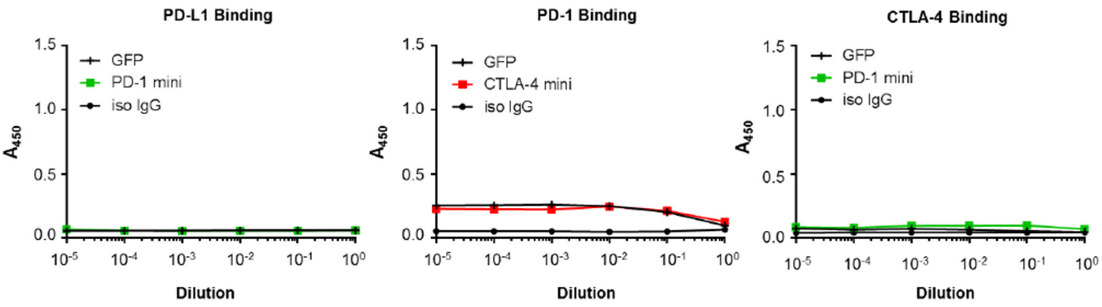

Figure S3. Minibody binding controls. A549 cells were transfected with GFP expression plasmid alongside minibody expression plasmids to serve as a control for minibody binding. Media containing GFP or an irrelevant minibody were assessed for binding to recombinant human protein (PD1minibody:PDL1, CTLA4minibody:PD1, PD1minibody:CTLA4). Isotype IgG were used as controls (10ug/mL; highest concentration).

A

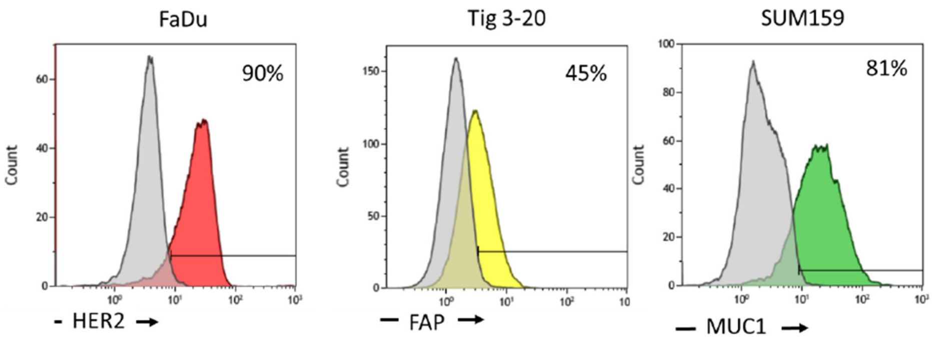

B

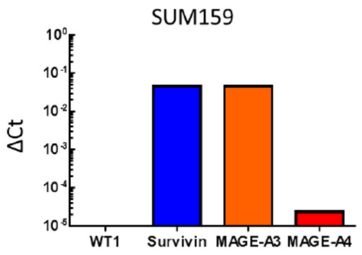

Figure S4. Tumor target antigen expression. (**A**) HER2, FAP, and MUC1 cell surface expression on FaDu, Tig-3-20, and SUM-159 cells respectively, were analyzed by flow cytometry. (**B**) RNA was extracted from SUM-159 cells and expression of the Cancer/Testis antigens WT1, Survivin, MAGE-A3, and MAGE-A4 was analyzed by qRT-PCR. Data were normalized with human B-actin.

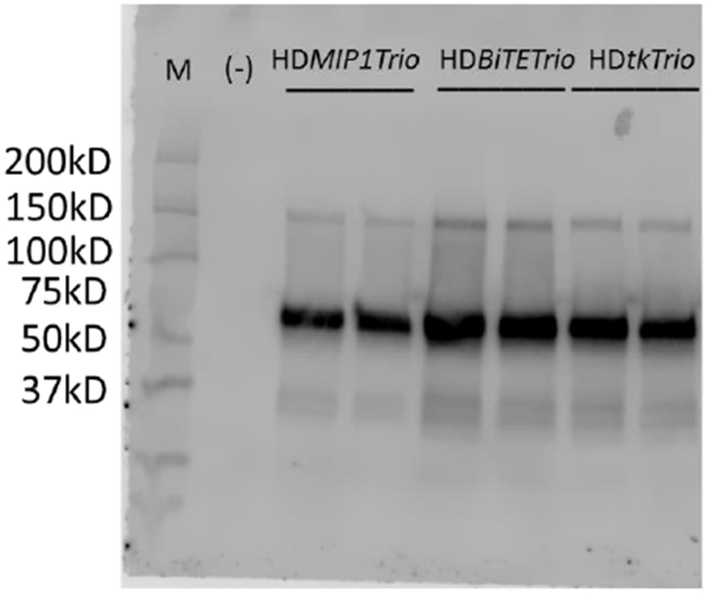

Figure S5. Complete, uncropped western blots for HD*Trios* minibody expression.

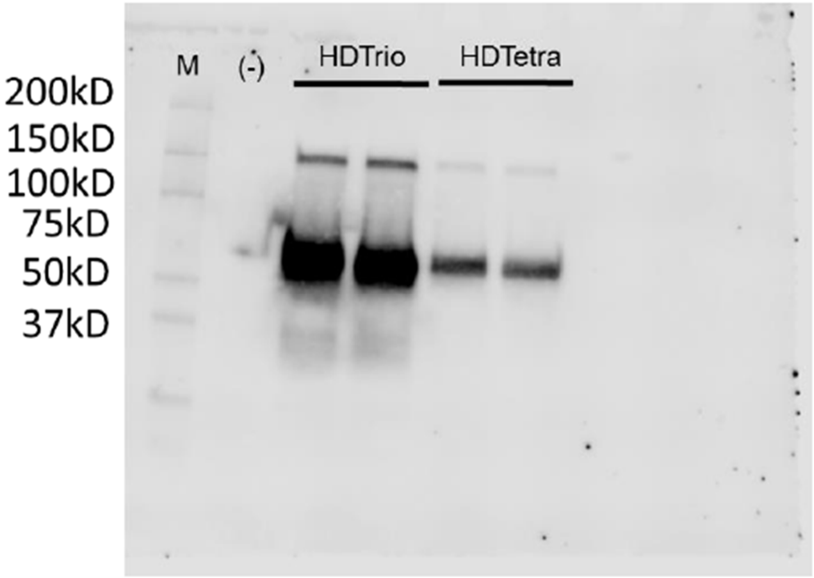

Figure S6. Complete, uncropped western blots for HD*Trio* and HD*Tetra* minibody expression.
